# A novel circular RNA, circXPO1, promotes lung adenocarcinoma progression by interacting with IGF2BP1

**DOI:** 10.1038/s41419-020-03237-8

**Published:** 2020-12-02

**Authors:** Qi Huang, Haifa Guo, Shaodong Wang, Yi Ma, Haiming Chen, Hao Li, Jiawei Li, Xiao Li, Fan Yang, Mantang Qiu, Song Zhao, Jun Wang

**Affiliations:** 1 Department of Thoracic Surgery, Peking University People’s Hospital, Beijing, 100044 China; 2 Department of Thoracic Surgery, The First Affiliated Hospital of Zhengzhou University, Zhengzhou, 450003 China; 3 Department of Thoracic Surgery, Beijing Chest Hospital, Capital Medical University, Beijing, 100095 China

**Keywords:** Non-small-cell lung cancer, Non-coding RNAs

## Abstract

Studies have demonstrated that noncoding RNAs play important roles in various types of cancer; however, noncoding RNAs derived from regions of genomic alterations have rarely been explored, especially for circular RNAs (circRNA). Previously, we found several circRNAs were upregulated in lung adenocarcinoma (LUAD) tumor tissues by RNA sequencing. Here, we characterized a novel circRNA, circXPO1, in LUAD, which is derived from a well-established cancer therapeutic target, XPO1. circXPO1, is formed by back-splicing of exon 3 and exon 4 of XPO1 gene. circXPO1 was highly expressed in LUAD tissues compared with paired adjacent non-tumor tissues, and high circXPO1 expression correlated with worse overall survival. circXPO1 expression was positively correlated with the XPO1 gene copy number. Mechanically, circXPO1 could bind with IGF2BP1 and enhance CTNNB1 mRNA stability, and subsequently promote LUAD progression. In a LUAD patient-derived xenograft model, intratumoural injection of cholesterol-conjugated siRNA specifically targeting circXPO1 efficiently suppressed tumor growth. To summary, these results suggest that circXPO1 is critical for LUAD progression and may serve as a biomarker for poor prognosis and a therapeutic target. On the other hand, the functional roles of noncoding transcripts derived from coding genes should be re-evaluated.

## Facts

- Copy number amplification contributes to circXPO1 overexpression in lung adenocarcinoma.
- circXPO1 high expression promotes lung adenocarcinoma progression by binding to IGF2BP1 and stabilizing CTNNB1 mRNA.
- High circXPO1 expression indicates poor survival of lung adenocarcinoma patients and might be a therapeutic target.

## Introduction

Lung cancer is the leading cause of tumor-related deaths worldwide. Lung adenocarcinoma (LUAD) is the major histological type of lung cancer, and the incidence of LUAD is increasing in China^1^. Despite the development of new therapeutic approaches for LUAD, the 5-year survival rate only ranges from less than 12 to 15%^2^. Therefore, to improve the therapeutic effects, further exploration of the molecular mechanisms and identification of proper biomarkers for LUAD are necessary.

Circular RNAs (circRNAs), as a unique class of noncoding RNAs (ncRNAs), have distinct properties and diverse functions across cell types and tissues in mammals^3,4^. circRNAs perform regulatory roles and exhibit the well-known “miRNA sponging” function in multiple diseases, including cancers^5,6^. In addition, circRNAs can interact with different RNA binding proteins (RBPs) to form specific circRNA-RBP complexes (circRNPs) that subsequently influence the modes of action of their associated proteins^7^; one example is circNSUN2, which can enhance the stability of HMGA2 mRNA by enhancing its interaction with IGF2BP2 to promote malignant progression of colorectal cancer^8^. To date, the contribution of RBPs to the underlying mechanism of circRNAs remains largely unknown.

Copy number alterations (CNAs) are somatic changes that cause the duplication or deletion of sections of the genome; these alterations affect tumor progression and metastasis and promote poor clinical outcomes in cancers^9,10^. Due to genomic instability, CNAs are prevalent in somatic tissues and are especially prevalent in numerous cancers, and they play indispensable roles in activating oncogenes and inactivating tumor suppressors in lung, breast, and pancreatic cancers^11–13^. Exploration of the biological and phenotypic effects of CNAs is meaningful in cancer diagnostics and therapeutics. In LUAD, high tumor grade and distant metastasis are positively associated with the proportion of subclonal CNAs^14^. Previous research indicated that the levels of circRNAs can be influenced by genetic alterations, such as translocations and CNAs^15^. However, CNA-associated circRNAs have rarely been detected in LUAD.

In our previous study, we identified a series of differentially expressed circRNAs in LUAD via ribosomal RNA-depleted RNA sequencing^16^. Here, we characterized a novel circRNA, circXPO1, derived from the XPO1 gene, that can promote LUAD progression and might serve as a therapeutic target. Further investigation indicated that circXPO1 can bind to IGF2BP1 and enhance the stability of CTNNB1 mRNA.

## Materials and Methods

### Cell lines

All cell lines (A549, SPC-A1, NCI-H460, NCI-H2126, NCI-H1703, NCI-H1299, and 16-HBE) were obtained from the Cell Bank of the Chinese Academy of Sciences (Shanghai, China). SPC-A1 cells were cultured in Dulbecco’s modified Eagle’s medium (DMEM, Gibco, 2125039), and others were cultured in RPMI 1640 medium (Gibco, 2122752). All media contained 10% foetal bovine serum (FBS). All cell lines were cultured at 37 °C with 5% CO_2_. The cell lines were identified by STR and tested for mycoplasma contamination.

### Patients and clinical samples

This study was approved by the Ethical Review Committee of Peking University People’s Hospital. LUAD tissues and paired non-tumor lung tissues were acquired from patients undergoing surgery in the Department of Thoracic Surgery, Peking University People’s Hospital. None of the patients included in this study underwent any preoperative radiation or chemotherapy. The clinical data and follow-up information were collected. The pathological analysis was based on the 8th TNM-classification of IASLC^17^. Written informed consent was obtained from each patient.

### RNA extraction, gDNA extraction, and qRT-PCR analysis

RNA extraction and qRT-PCR were performed, as described previously^18^. Genomic DNA (gDNA) was extracted from cultured cells according to the PureLink Genomic DNA Mini Kit protocol (Thermo Fisher Scientific, K182001). GAPDH, ACTB, and ACTR3BP2 were used as internal controls, and the expression level of circXPO1 was measured by qRT-PCR. The primers used in this study are listed in Table S1. The relative gene expression levels were normalized to those of the internal control, and the 2^−^^ΔΔCt^ method was used to calculate the fold changes in expression (tumor vs. normal).

### Nucleic acid electrophoresis

The complementary DNA (cDNA) and gDNA PCR products were analyzed by 4% agarose gel electrophoresis with TBE running buffer. DNA was separated by electrophoresis at 110 V for 30 min. DL500 (KeyGEN, KGM003) was used as the DNA marker. The bands were visualized by UV irradiation.

### RNA isolation of nuclear and cytoplasmic fractions

The subcellular localization of circXPO1 was investigated using a PARIS Kit according to the manufacturer’s protocol (Ambion, AM1921).

### MS2-MBP–mediated pull-down of circXPO1-bound proteins

RBPs associated with circXPO1 were identified using the maltose-binding protein (MBP) affinity purification method. The MS2-MBP protein was expressed in *E. coli* and purified in accordance with a protocol from the Steitz laboratory. Three bacteriophage MS2 coat protein-binding sites (5′-cgtacaccatcagggtacgagctagcccatggcgtacaccatcagggtacgactagtagatctcgtacaccatcagggtacg-3′) were inserted downstream of circXPO1 by site-directed mutagenesis with a Stratagene QuikChange Site-Directed Mutagenesis Kit. To obtain RBPs associated with circXPO1, A549 cells were transfected with 50 μg MS2-tagged circXPO1 constructs, and 1 × 10^7^ cells were used for each RNA pull-down assay. Cells were harvested 48 h post-transfection and subjected to the RNA pull-down assay^19^.

### RNA immunoprecipitation

An EZ Magna RIP Kit (Millipore, 17-701) was used in accordance with the manufacturer’s protocol. Ten million A549 cells were lysed in complete RNA immunoprecipitation (RIP) lysis buffer (150 mM KCl, 25 mM Tris pH = 7.4, 0.5 mM DTT, 0.5% NP40, 1 mM PMSF and a protease inhibitor) for 5 min at 4 °C, and the cell extract was incubated with magnetic beads conjugated with anti-IGF2BP1 (AB_1570640) or control anti-IgG antibody (AB_843202) for 6 h at 4 °C. The beads were incubated with proteinase K(1.2 mg/ml) at 55 °C for 30 min to separate the protein components, leaving the RNAs intact. Finally, purified RNA was subjected to qRT-PCR analysis.

### Assessment of endogenous RNA degradation

Cells were incubated with 5 mg/ml actinomycin D (Sigma, A4262) and collected in TRIzol at the indicated time points after treatment, as described previously^20^. CircXPO1 was specifically silenced using siRNA in SPC-A1 and was overexpressed by transfecting circXPO1-vector in A549 cell lines. The cells were incubated with actinomycin D for 0 min, 30 min, 1 h, 2 h, 4 h, 8 h,12 h, and 24 h, respectively, and then were extracted for RNA extraction. The initial cell number is about 8 × 10^4^ cells. Purified RNA was reverse transcribed to cDNA and analyzed by qRT-PCR as described above.

### Construction of small interfering RNAs (siRNAs) and plasmids and transfection of cells

circXPO1 was specifically silenced using siRNA targeting the circXPO1 back-splice junction. The siRNA construct was designed with CircInteractome (https://omictools.com/circinteractome-tool) and synthesized by GenePharma (Shanghai, China). Full-length human circXPO1 cDNA was synthesized by Invitrogen and cloned into the expression vector pCDNA3.1 (GENERAY BioTECH, Shanghai). The final construct was verified by sequencing. LUAD cells were seeded in 6-well plates and 24 h later, at a confluence of 60-70%, were transfected with specific siRNA (100 nM) or control siRNA (100 nM) using Lipofectamine® RNAiMAX according to the manufacturer’s protocol (Invitrogen;13778100). Plasmid vectors for transfection were prepared in Opti-MEM (Gibco,31985070) and transfected into LUAD cells using X-tremeGENE HP DNA Transfection Reagent (Roche, 6366236001). All siRNA sequences used are listed in Table S1.

### Cell proliferation, invasion, and apoptosis assays

Cell proliferation was evaluated using an MTT Kit (KeyGEN, KGA317S) and a 5-ethynyl-2′-deoxyuridine (EdU) assay (KeyGEN, KGA331-100) in accordance with the protocols provided by the manufacturer. Cells were incubated in a normal culture medium containing 10% MTT. The proliferation rate was determined at 0 h, 24 h, 48 h, 72 h, and 96 h after transfection. The absorbance of each well was measured with a microplate reader set at 490 nm. Colony formation assays were performed to evaluate the clonogenic ability of the cells. Transwell assays and wound healing assays were performed to evaluate the migration and invasion capabilities, respectively. Cells were grown in 24-well plastic dishes and treated with circXPO1 siRNA or vector. After 24 h transfection, 5 × 10^4^ cells in serum-free medium were seeded in the transwell migration chambers (Millipore, PIEP12R48). Medium containing 10% FBS was added to the lower chambers. Migrated and invaded cells were stained with crystal violet and were then counted using a light microscope. Each experiment was performed in triplicate.

### In vivo tumor growth assays

All animal experiments were conducted in accordance with relevant guidelines and were approved by the Animal Care Committee of Peking University People’s Hospital. Female BALB/c nude mice (4–6 weeks, 18–20 g) were purchased from Charles River (Beijing, China) and were bred and maintained under specific pathogen-free conditions. Mice were randomly assigned to negative control and si-circXPO1 groups. Negative control and si-circXPO1-transfected SPC-A1 cells were harvested. For the tumor formation assay, 1 × 10^6^ cells/mouse were subcutaneously injected into one flank of each mouse. Tumor growth was inspected weekly using a straightedge, and the tumor volume was calculated using the standard equation V = 0.5 × D × d^2^ (V, volume; D, longitudinal diameter; d, transverse diameter).

### In vivo human PDX model-based therapeutic study

Patient-derived xenograft (PDX) models were established to evaluate the clinical therapeutic benefit of targeting circXPO1. PDX models were established as described in a previous publication^21^. Fresh tissues were sliced into 1–3 mm^3^ fragments and subcutaneously implanted into the flank of mice. Animals were anaesthetized with 10 mg/kg Zoletil and 3 mg/kg Rompun by intraperitoneal injection before tumor implantation (*n* = 5 per tumor sample). When the tumors had grown to palpable size (~200 mm^3^), the tumor-bearing mice were randomly divided into two experimental groups. One hundred microlitres of cholesterol-modified control siRNA or si-circXPO1 (2 OD) dissolved in distilled water was intratumourally injected twice weekly for two weeks. At the end of the experiment, the excised xenografts were freeze dried in nitrogen for further analysis, and one fragment was fixed with 10% neutral buffered formalin for histologic examination.

### Western blot analysis

Cells were lysed with RIPA buffer (Cell Signalling Technology, 9806 S) containing a protease inhibitor and phosphatase inhibitor cocktail (Sigma, MSSAFE-1VL). The concentration of the extracted protein was measured using the BCA method, and samples were subjected to SDS-PAGE. Proteins were transferred to polyvinylidene fluoride membranes (Millipore, SAMP2GVNB), which were blocked with 5.0% non-fat milk in TBS for 1 h at room temperature and incubated with primary antibodies overnight at 4 °C. Membranes were washed three times with TBST and incubated with the corresponding HRP-conjugated secondary antibodies for 2 h at room temperature. Protein detection was performed using a chemiluminescence system (Bio-Rad, USA). The antibodies for Western blotting were listed in Table S2.

### Statistical analysis

All quantitative data are presented as the means ± standard deviations of data from at least three independent experiments. Differences between groups were analyzed by a paired two-tailed *t* test. One-way analysis of variance (ANOVA) or the nonparametric Kruskal-Wallis test was used to evaluate the associations between circXPO1 expression and other characteristics. The strength of the association between continuous variables was analyzed by Spearman correlation analysis. The levels of significance were set at *p < 0.05 and **p < 0.01. The prognostic value of circXPO1 expression was further analyzed using the Kaplan-Meier method. All statistical analyses were performed using SPSS 20.0 software (Armonk, New York; IBM Corp).

## Results

### circXPO1 is a novel circRNA transcript in LUAD

**Fig. 1:**
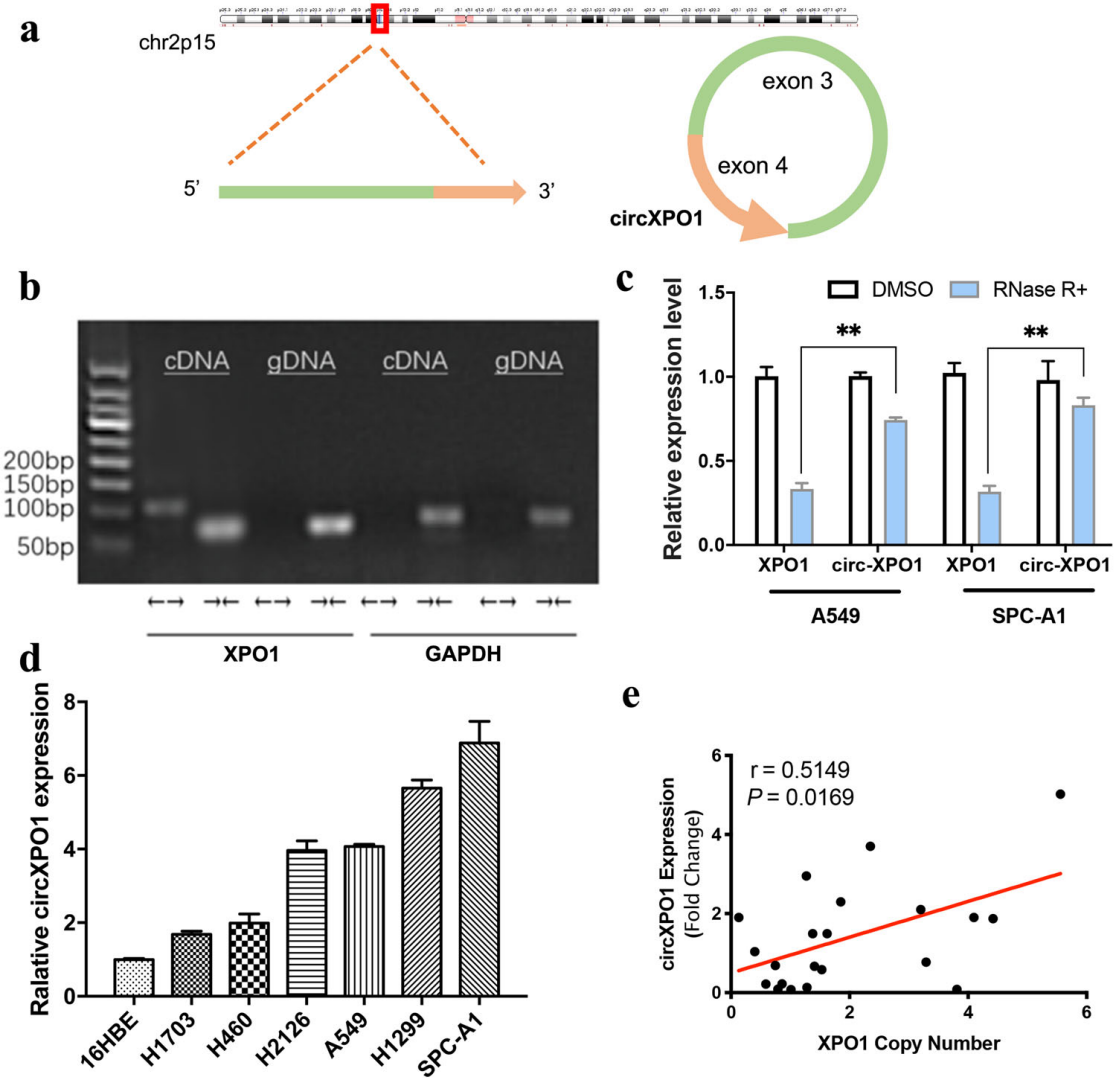
Circular RNA transcript of the XPO1 gene. (**a**) Localization of circXPO1 and back-splicing of exon 3 and exon 4. (**b**) Agarose gel electrophoresis analysis of PCR products of divergent primer and convergent primer. ←→ divergent primers; →← convergent primers. GAPDH was used as a control for a linear RNA transcript. cDNA: complementary DNA, gDNA: genomic DNA. (**c**) Relative expression level of XPO1 and circXPO1 in A549 and SPC-A1 after RNase R digestion. DMSO treated cells were used as a control and set to 1. *P < 0.05, **P < 0.01, *P* values were calculated with Mann–Whitney U test. (**d**) Expression of circXPO1 in normal lung cells (HBE) and lung cancer cell lines. HBE was used as a reference and set to 1. (**e**) Dot plot of XPO1 gene copy number and circXPO1 gene expression. *P* value was calculated by Spearman correlation.

In our previous work^16,22^, we identified many differentially expressed circRNAs in LUAD and found that copy number variation (CNV) of oncogenes may alter circRNA expression. We observed that a circular transcript of XPO1 (circBase ID: hsa_circ_0001016, denoted “circXPO1” in this study) is highly expressed in LUAD tissues compared with adjacent non-tumor tissues. circXPO1 is a 175 nt circRNA transcript generated by back-splicing of exon 3 and exon 4 of the XPO1 gene (Fig. 1a). Divergent primers (to detect circular transcripts) and convergent primers (to detect linear transcripts) were designed, and the convergent primers were found to amplify only cDNA, confirming the circular structure of the RNA transcript (Fig. 1b). Sanger sequencing of PCR products also demonstrated the back-splice junction site (shown in our previous work^16^). Compared with the linear XPO1 RNA transcript, circXPO1 is resistant to RNase digestion (Fig. 1c). Additionally, circXPO1 is highly expressed in LUAD cell lines compared with the human bronchial epithelial cell line 16HBE (Fig. 1d). We further analyzed the association between the circXPO1 level and XPO1 gene copy number in LUAD tumor tissues. To this end, we set an independent cohort of 21 LUAD patients and extracted both gDNA and RNA from tumor tissues and adjacent normal tissues. XPO1 CNV was detected in gDNA, with ACTR3BP2, which is located on the same chromosome but close to the centromere, as the internal control. XPO1 exhibited a copy number gain in LUAD tumor tissues, and we observed a significant positive correlation between the XPO1 gene copy number and circXPO1 expression level (r = 0.515, *P* = 0.017 for Spearman correlation; Fig. 1e). This result indicated that high expression of circXPO1 might be caused by copy number gain of the XPO1 gene. Thus, we identified a novel circRNA, circXPO1, derived from the XPO1 gene, in LUAD tissue.

### Correlation between circXPO1 expression and clinical characteristics of LUAD

**Fig. 2:**
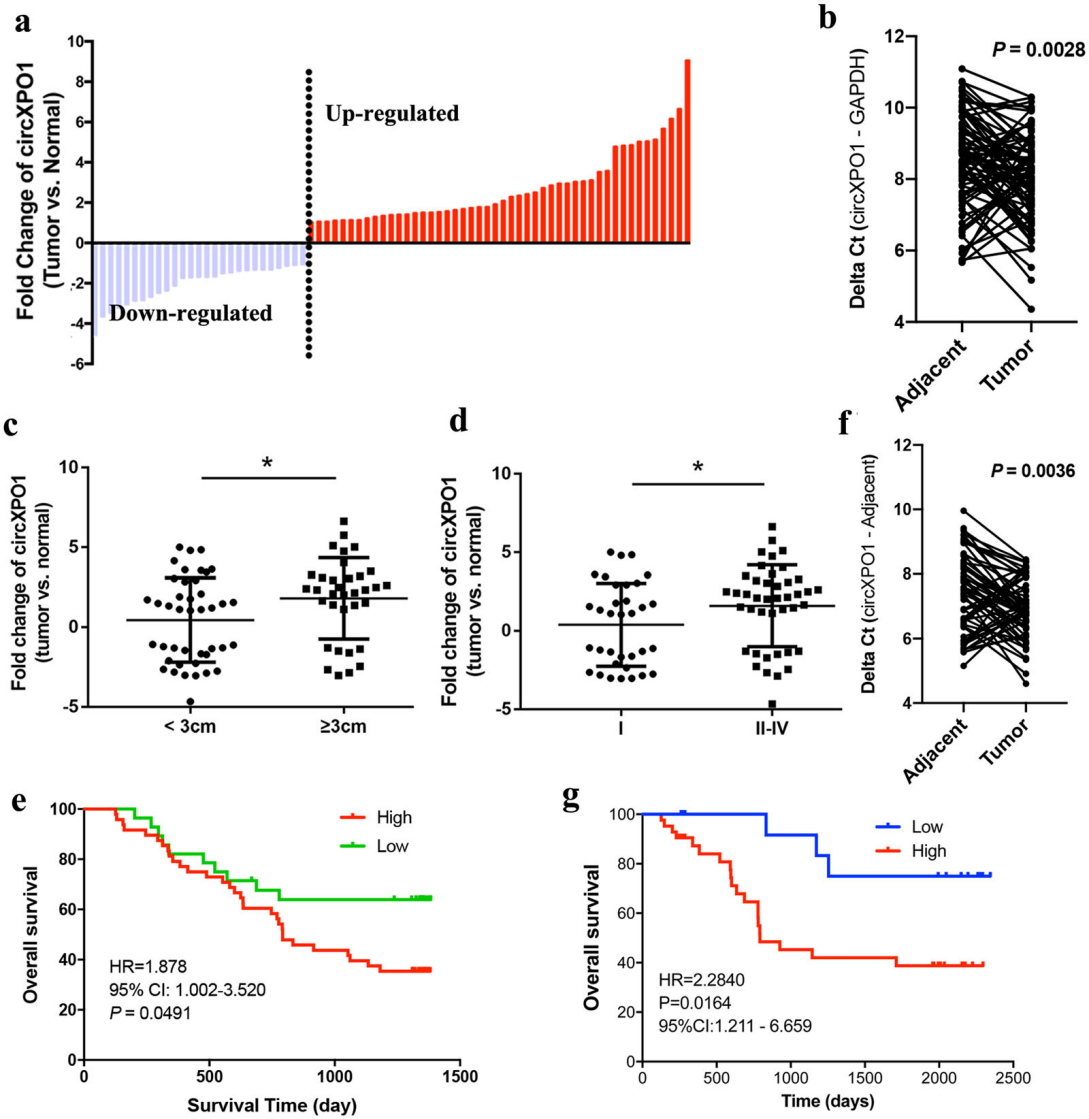
High circXPO1 expression is associated with LUAD stage and survival. (**a**, **b**) circXPO1 expression in 76 pairs of LUAD tumor tissues and adjacent lung tissues measured by qRT-PCR. (**c**) The circXPO1 expression level in patients with tumors ≥3 cm and <3 cm (non-paired *student* test was performed). (**d**) The circXPO1 expression level in patients with different TNM stages (non-paired *student* test was performed). (**e**) Kaplan–Meier analysis of circXPO1 in 76 LUAD patients (HR = 1.878, 95% CI: 1.002–3.52, *P* = 0.0491 with the Kaplan–Meier method). (**f**, **g**) Kaplan-Meier analysis of circXPO1 in another cohort of 61 LUAD patient (HR = 2.28, 95% CI: 1.21–6.66, *P* = 0.016 with the Kaplan–Meier method). CircXPO1 expression was classified by mean fold change (tumor vs. normal) in **d** and **e**.

To explore the clinical relevance of circXPO1 expression in patients with LUAD, we further collected 76 pairs of primary LUAD tumor tissues and paired adjacent non-tumor tissues. Via qRT-PCR, we found that circXPO1 was significantly overexpressed in LUAD tumor tissues compared with matched adjacent non-tumor tissues (Fig. 2a, b). In addition, the expression level of circXPO1 was significantly higher in patients with larger tumors and a more advanced TNM stage (Fig. 2c, d). Survival analysis revealed that patients with high expression of circXPO1 had shorter overall survival times (HR = 1.88, 95% CI: 1.002–3.52, *P* = 0.0491; Fig. 2e), suggesting that high expression of circXPO1 might be a biomarker for poor survival. To validate the prognostic role of circXPO1, we detected circXPO1 expression in an independent cohort of 61 LUAD patients. In this cohort, circXPO1 was also significantly upregulated in LUAD tumor tissues (Fig. 2f) and high expression of circXPO1 was associated with shorter overall survival times (HR = 2.28, 95% CI: 1.21–6.66, *P* = 0.016; Fig. 2g). However, multivariate Cox-regression showed circXPO1 was not an independent prognostic factor in both cohorts (Table S3 and S4). These lines of evidence confirm that circXPO1 is highly expressed in LUAD tumor tissues and that higher circXPO1 expression indicates a poorer prognosis.

### CircXPO1 promotes the proliferation and invasion of LUAD cells in vitro

**Fig. 3:**
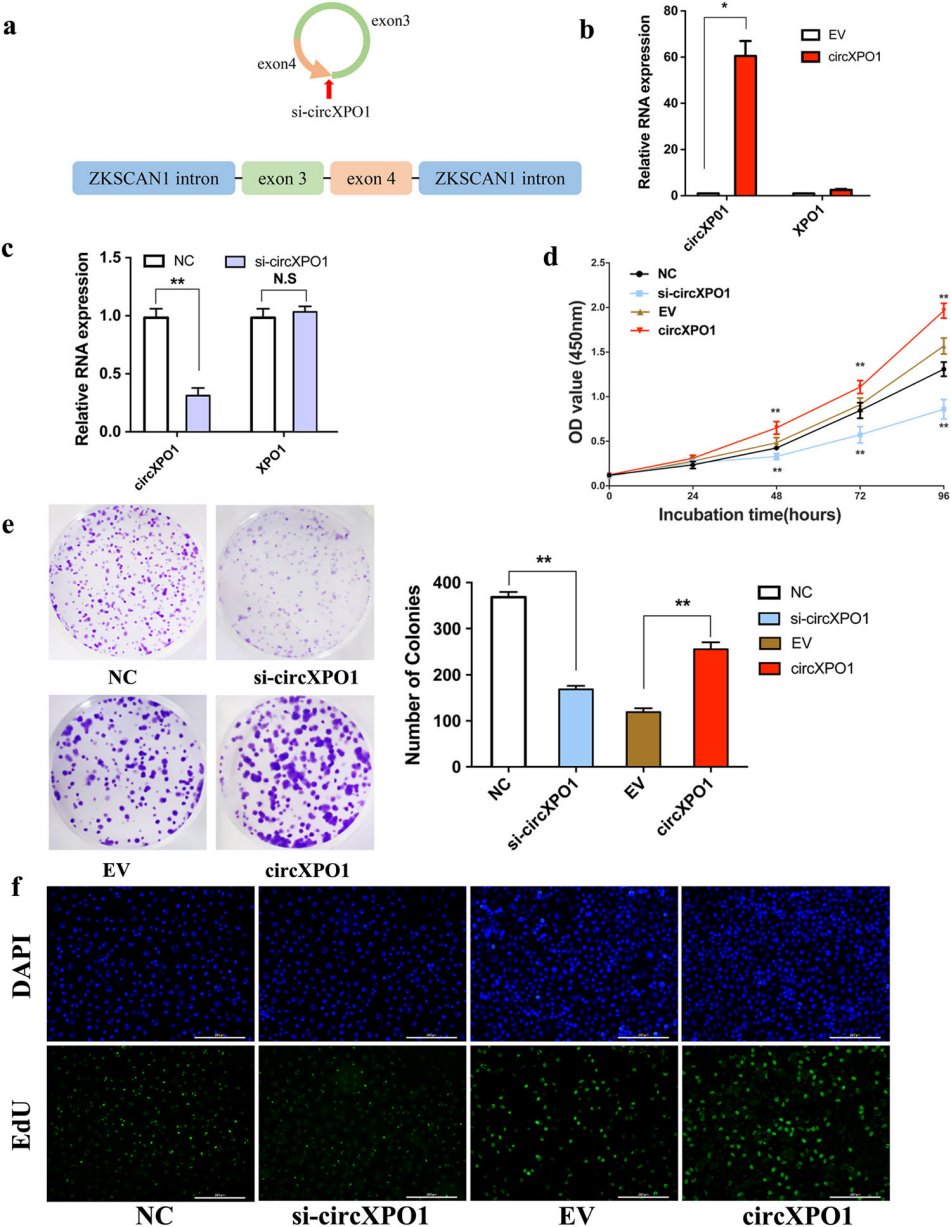
circXPO1 promotes the proliferation of LUAD cells. (**a**) Schematic illustration showing the siRNA specifically targeting the circXPO1 splice junction and the overexpression vector. qRT-PCR analysis of circXPO1 expression and XPO1 mRNA expression after transfection of the expression vector (**b**) and siRNA (**c**). MTT (**d**), colony formation (**e**), and EdU (**e**) assays in SPC-A1 cells after silence or overexpression of circXPO1. The scale bars represent 200 µm in **e**. (*P* values were calculated by non-paired student tests. *P < 0.05, **P < 0.01. The error bars indicate the standard deviations.).

To investigate the functional role of circXPO1 in LUAD carcinogenesis, we designed a siRNA and an expression vector for silencing and overexpression of circXPO1, respectively. The siRNA was designed to specifically target the circXPO1 back-splice junction (Fig. 3a). For ectopic overexpression of circXPO1, exon 3 and exon 4 of XPO1 were cloned into an expression vector, as previously reported^23^. In A549 cells, transfection of the expression vector led to an increase of approximately 60-fold in circXPO1 expression compared with that in cells transfected with empty vector (Fig. 3b). Additionally, compared with the negative control siRNA, si-circXPO1 greatly decreased the circXPO1 level in SPC-A1 cells but did not affect the level of linear XPO1 (Fig. 3c).

Silencing of circXPO1 significantly suppressed the proliferation ability of SPC-A1 cells, whereas overexpression of circXPO1 promoted A549 cell proliferation, as revealed by the results of MTT, colony formation, and EdU proliferation assays (Fig. 3d-f). The TUNEL assay results showed that silencing of circXPO1 increased the percentage of apoptotic cells, while ectopic expression of circXPO1 decreased apoptosis (Fig. S1a). Furthermore, silencing circXPO1 significantly decreased but circXPO1 overexpression increased the metastatic ability of LUAD cells (Fig. S1b and S1c). Notably, we also found that the markers of cell proliferation (PCNA) and migration (E-cadherin and Vimentin) also changed accordingly in A549 cells (Fig. S1d). Taken together, these results demonstrate that circXPO1 can promote the proliferation and migration of LUAD cells.

### circXPO1 increases CTNNB1 mRNA stability by interacting with IGF2BP1

**Fig. 4:**
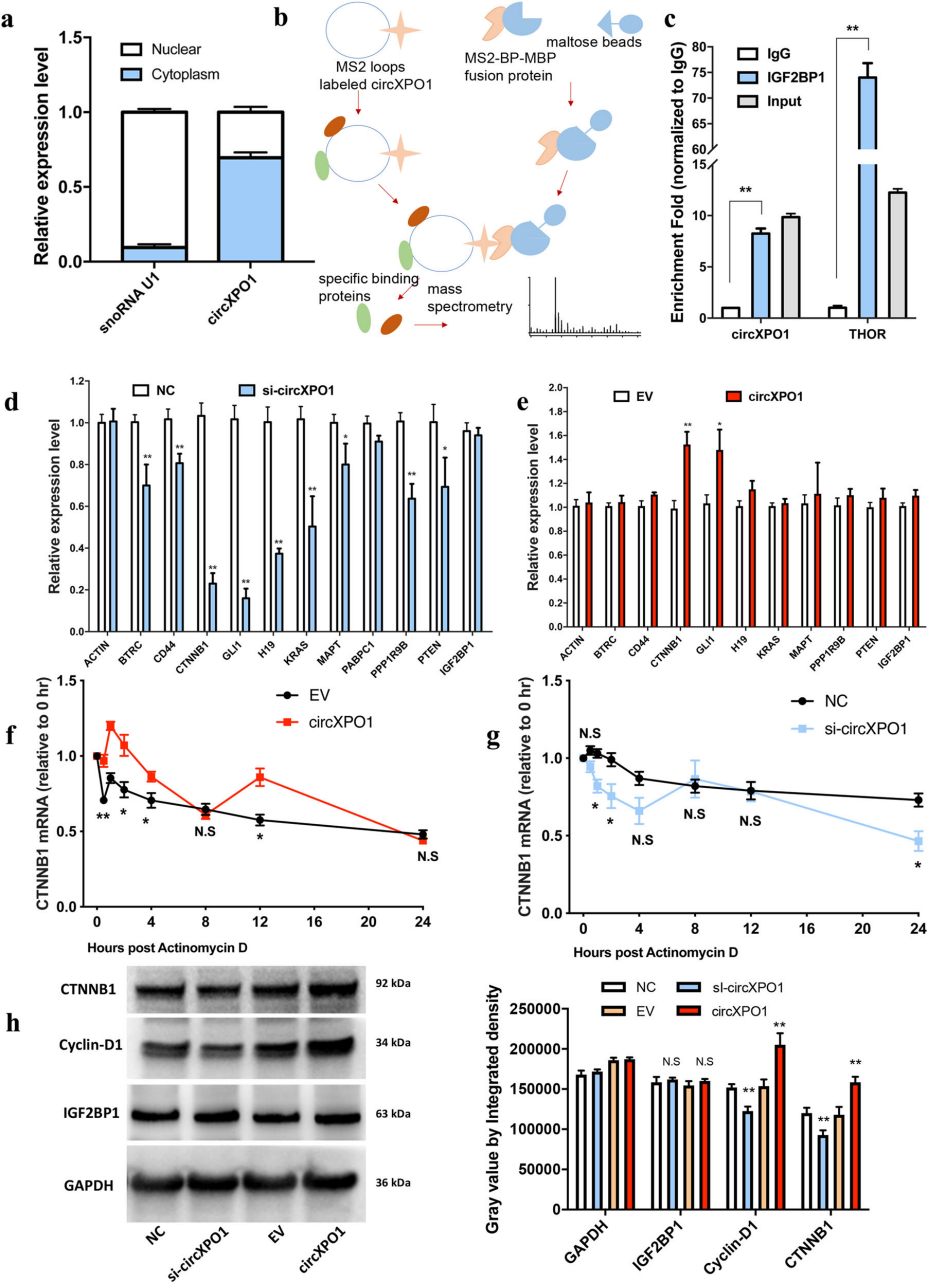
circXPO1 binds to IGF2BP1 and enhances CTNNB1 mRNA stability. (**a**) Subcellular location of circXPO1 in A549 cells. (**b**) Flow chart of the MS2 pull-down assay protocol. (**c**) RNA immunoprecipitation assay using the anti-IGF2BP1 antibody in A549 cells. lncRNA THOR is used as a positive control of IGF2BP1-binding RNA. Canonical target mRNAs of IGF2BP1 were detected by qRT-PCR after silence (**d**) and overexpression of (**e**). qRT-PCR was used to measure the expression levels of CTNNB1 after actinomycin D treatment in the context of circXPO1 overexpression (**f**) and (**g**) silence in A549 cells. (**h**) Protein expression of circXPO1 downstream targets upon silence and overexpression in A549 cells. (*P* values were calculated by non-paired student tests. *P < 0.05, **P < 0.01. The error bars indicate the standard deviations.).

To further explore the molecular mechanisms of circXPO1 in LUAD pathogenesis, we first analyzed the subcellular location of circXPO1. The results of qRT-PCR analysis after cell fractionation suggested that circXPO1 is predominantly localized in the cytoplasm (Fig. 4a). The small nucleolar RNA (snoRNA) U1 was selected as the classical positive marker of the cell nucleus because it is localized in the nucleus and has stable expression. circRNAs can interact with various RBPs to form specific circRNA-RBP complexes that subsequently influence the modes of action of their associated proteins^7^. To identify the RBPs that may bind to circXPO1, we performed an MS2 RNA pull-down assay. We constructed a vector expressing circXPO1, which was labeled with MS2 (V_circXPO1-MS2_), and a vector expressing only MS2 (V_MS2_). By labeling circXPO1 with the MS2 structure, we were able to use beads conjugated with the MS2-binding protein to pull down RBPs that interact with circXPO1 (Fig. 4b). Mass spectrometry showed that IGF2BP1 was the most abundant protein pulled down by V_circXPO1-MS2_ and that IGF2BP1 was not pulled down by V_MS2_ (Table S5). Furthermore, to confirm the interaction between IGF2BP1 and circXPO1, we performed a RIP assay using an anti-IGF2BP1 antibody (Fig. 4c). Lysates harvested from A549 cells were incubated with the anti-IGF2BP1 antibody or IgG in accordance with the RIP protocol, and the precipitated RNA was analyzed by qRT-PCR. The lncRNA THOR was used as the positive control since it has been demonstrated to bind to IGF2BP1^20^. As shown in Fig. 4c, circXPO1 transcripts were specifically enriched by the anti-IGF2BP1 antibody in A549 cells. Thus, these findings demonstrate that circXPO1 can bind to IGF2BP1.

IGF2BP1 is a widely expressed RBP that is a core compartment of the messenger ribonucleoprotein (mRNP) complex. Studies have reported that IGF2BP1 can modulate the mRNA stability of a series of well-elucidated target RNAs (IGF2, CD44, KRAS, ACTB, PABPC1, GLI1, MYC, MAPT, CTNNB1, PPP1R9B, BTRC, PTEN, and H19)^20^. In addition, recent reports have shown that several ncRNAs can modulate mRNA stability by interacting with IGF2BP1, such as the lncRNA THOR^24^. Thus, we speculated that circXPO1 might regulate the mRNA stability of IGF2BP1’s target mRNA. To further investigate the downstream genes of circXPO1, we measured the mRNA levels of IGF2BP1 target genes and found that the mRNA level of CTNNB1 was significantly decreased upon silencing of circXPO1 and increased upon overexpression of circXPO1 (Fig. 4d, e).

CTNNB1 (also called β-Catenin) is an integral transcription factor in canonical Wingless-Int (Wnt) signalling and is an essential oncogene implicated in key cellular functions during tumourigenesis and malignant progression, including in lung cancer^25–27^. Suppression of CTNNB1 downregulates its downstream transcriptional targets (c-Myc, Cyclin D1, and CDK4) and suppresses the proliferation of lung cancer cells^28^. Thus, we hypothesized that circXPO1 might enhance the expression of CTNNB1 via mRNA stabilization mediated by the circXPO1-IGF2BP1 interaction. To corroborate this hypothesis, we used qRT-PCR to assess the mRNA stability of CTNNB1 at different times after actinomycin D treatment in the context of circXPO1 overexpression or silencing. Ectopic expression of circXPO1 increased but silencing of circXPO1 decreased CTNNB1 mRNA stability in A549 cells (Fig. 4f, g). Notably, we also found that overexpression of circXPO1 increased CTNNB1 mRNA stability and silencing circXPO1 decreased CTNNB1 mRNA stability in SPC-A1 cells (Fig. S2). In addition, silencing of circXPO1 decreased but overexpression of circXPO1 increased CTNNB1 protein expression in A549 cells (Fig. 4h). Additionally, the protein level of the CTNNB1 downstream target gene Cyclin D1 was altered accordingly (Fig. 4h).

Taken together, these data demonstrate that circXPO1, through its interaction with IGF2BP1, can enhance the stabilization of CTNNB1 mRNA in LUAD cells.

### circXPO1 promotes LUAD progression via the circXPO1/IGF2BP1-CTNNB1 axis

**Fig. 5:**
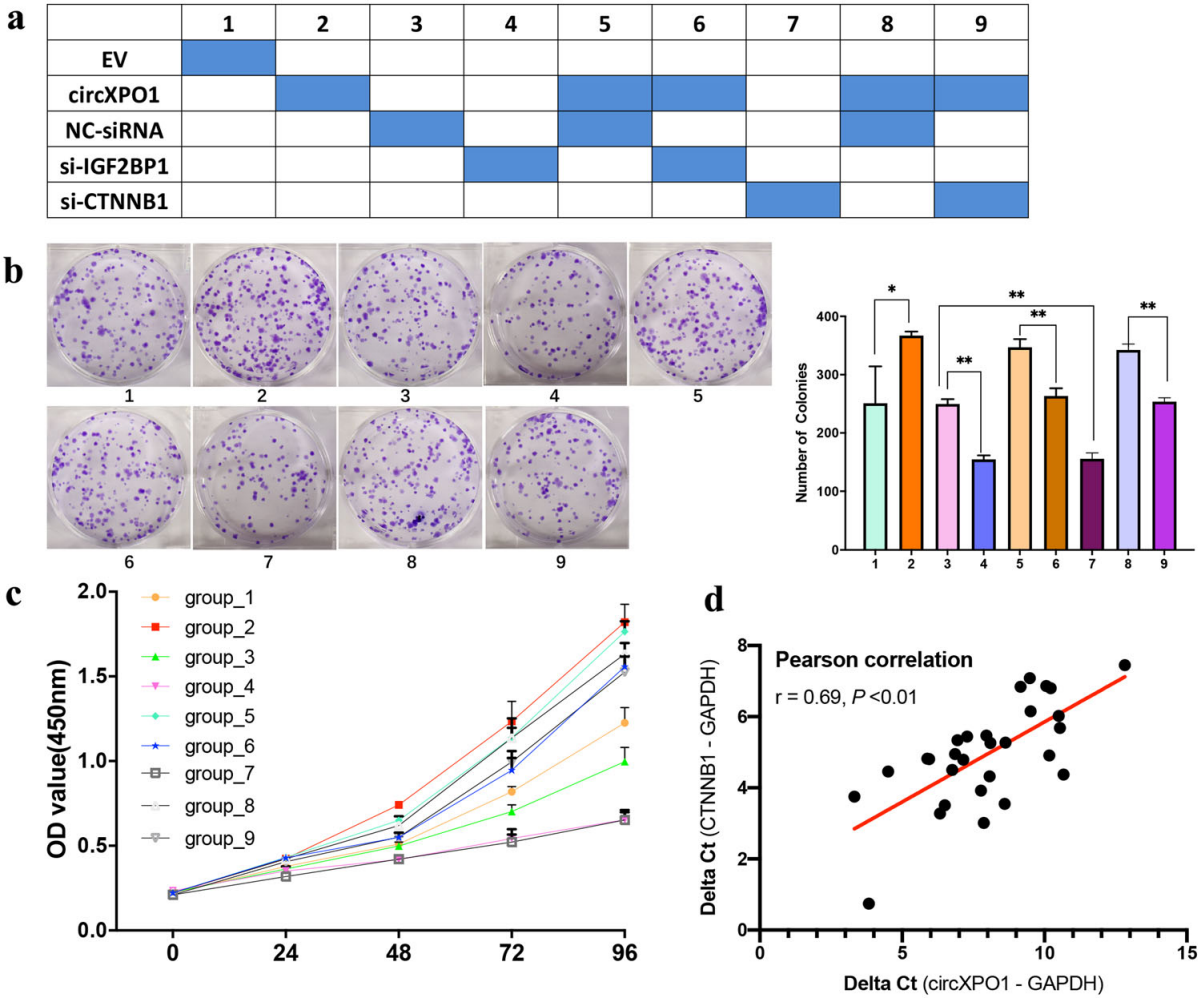
circXPO1 promotes LUAD progression via the circXPO1/IGF2BP1-CTNNB1 axis. Design of rescue experiments (**a**). Colony formation (**b**) and MTT assay (**c**) in A549 cells. (*P* values were calculated by non-paired student tests. *P < 0.05, **P < 0.01. The error bars indicate the standard deviations.) Dox plot of circXPO1 and CTNNB1 expression in LUAD tumor tissues (**d**).

We then inferred that circXPO1 might promote LUAD cell progression via the circXPO1/IGF2BP1-CTNNB1 axis. We designed functional rescue experiments in which we overexpressed circXPO1 and then silenced CTNNB1 and IGF2BP1 in A549 cells (Fig. 5a). As shown by the colony formation and MTT (Fig. 5b, c) assay results, overexpressing circXPO1 in A549 cells stimulated cancer cell proliferation, and the proliferative advantage conferred by circXPO1 overexpression was partially reversed by CTNNB1 silencing and IGF2BP1 silencing. These results were also found in SPC-A1 cells (Fig. S3). Furthermore, qRT-PCR results showed that circXPO1 expression level was positively correlated with CTNNB1 in LUAD tumor tissues (*r* = 0.69, *P* < 0.01; Fig. 5d). This evidence suggests that circXPO1 can promote LUAD cell proliferation via the circXPO1/IGF2BP1-CTNNB1 axis.

### circXPO1 promotes LUAD growth in vivo and serves as a prospective therapeutic target

**Fig. 6:**
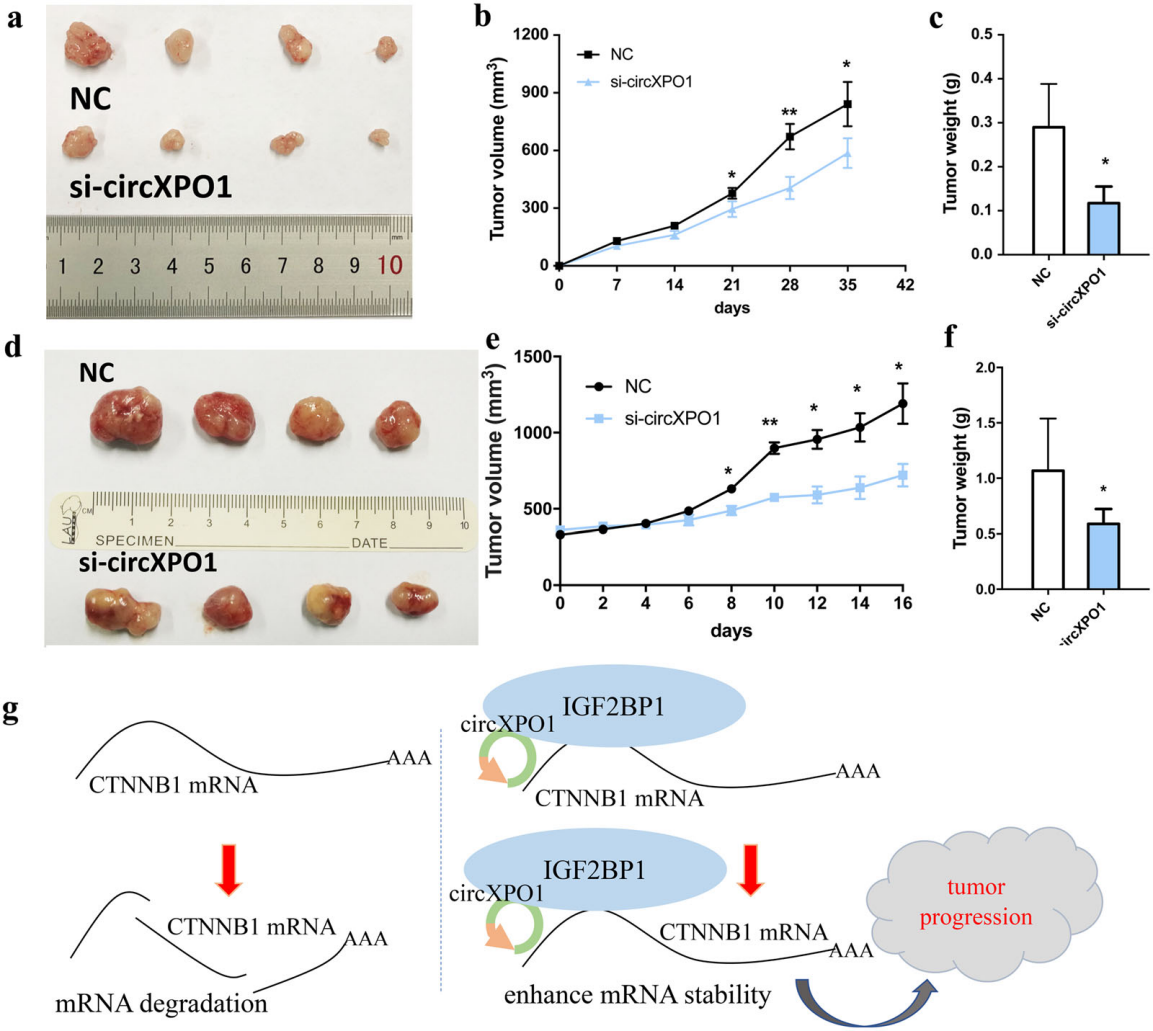
circXPO1 promotes LUAD tumor growth in vivo and is a potential therapeutic target. Xenograft tumor models were established using SPC-A1 cells transfected with negative control siRNA and si-circXPO1. Tumor size (**a**), growth curve of tumor volume (**b**), and tumor weight (**c**) were accessed for each derived xenograft tumor. Patient-derived xenograft (PDX) models were established and each tumor was intratumourally injected with negative control siRNA or si-circXPO1. Tumor size (**d**), growth curve of tumor volume (**d**), and tumor weight (**f**) were accessed for each tumor. Schematic diagram of the role of circXPO1 role in LUAD (**f**). XPO1 gene amplification leads to increased expression of circXPO1, and circXPO1 binds to IGF2BP1, enhancing CTNNB1 mRNA stability, thus increasing the CTNNB1 repression and promoting LUAD progression. (*P* values were calculated by non-paired student tests. *P < 0.05, **P < 0.01. The error bars indicate the standard deviations.).

To further evaluate the oncogenic role of circXPO1 in vivo, we established a xenograft tumor model in nude mice. SPC-A1 cells transfected with si-circXPO1 or negative control siRNAs were subcutaneously injected into BALB/c nude mice. A significant decrease in the size and weight of tumors from mice injected with si-circXPO1 compared to those from mice injected with control cells was observed (Fig. 6a–c). Immunohistochemical staining of xenograft tumor tissues indicated that tumor tissues from mice in the si-circXPO1 group contained fewer CTNNB1- and cyclin D1-positive cells than those from mice in the negative control group (Fig. S4).

PDX models specifically reflect the patient’s tumor heterogeneity and diversity, providing an essential oncology research platform on which to investigate the molecular mechanisms of tumor growth and predict the response to anticancer treatment^29^. We then established a PDX model with fresh LUAD tissue and evaluated the effect of circXPO1-targeted cancer therapy via intratumoural injection of cholesterol-conjugated si-circXPO1 or control siRNA (twice weekly, for a total of 4 times). circXPO1-targeted treatment significantly suppressed tumor growth in PDX model mice (Fig. 6d–f). The findings from these in vivo models suggest that circXPO1 silencing inhibits tumor growth and that circXPO1 may be a therapeutic target in LUAD.

## Discussion

In this work, we first revealed that circXPO1 is a novel circRNA located in the 2p15 amplicon that is frequently overexpressed in LUAD tumor tissues and showed that the expression of circXPO1 is positively correlated with advanced stage and shorter overall survival time in patients with LUAD. Next, the results of our mechanistic study elucidated that overexpressed circXPO1 in the cytoplasm binds to IGF2BP1 and enhances CTNNB1 mRNA stabilization to promote LUAD progression. Last, intratumoural injection of cholesterol-conjugated siRNA specifically targeting circXPO1 markedly suppressed the tumor growth in a PDX model. Considering that CTNNB1 is an essential transcription factor in canonical Wnt signalling^30^, our results highlight the clinical significance of circXPO1 as a novel therapeutic target in LUAD.

Among the differentially expressed circRNAs in LUAD revealed by ribosomal RNA-depleted RNA sequencing^16^, we characterized a novel circRNA, circXPO1, that is upregulated in LUAD and can promote LUAD progression. circXPO1 originates from exons 3 and 4 of its host gene XPO1. XPO1, also called chromosomal maintenance 1 (CRM1), is a well-known proto-oncogene involved in the development and progression of human tumors^31–35^. XPO1 mediates the nuclear export of various proteins, especially oncoproteins and tumor suppressor proteins, for example, p53^36^, BRCA1^37^, and p21^38^. We previously reported that circPRKCI is highly expressed in LUAD due to copy number gain and that circPRKCI can adsorb miR-589 and miR-545 to promote tumor cell proliferation via inhibition of their capability for suppressing E2F7^22^. To investigate whether circXPO1 is generated from amplified genomic regions, we evaluated the association between upregulated circXPO1 expression and XPO1 amplification. qRT-PCR data verified that genomic amplification of XPO1 was positively correlated with the expression level of circXPO1 expression. High expression of circXPO1 and its host gene XPO1 indicated that aberrant oncogene amplification results in overexpression of circular transcripts, a concept that may provide unconventional insight into our understanding of CNAs in tumorigenesis. Of note, copy number amplification is not the only reason for circXPO1 upregulation in LUAD, aberrant histone modification may also activate circXPO1 and linear XPO1 expression.

To date, most circRNAs have been linked with post-transcriptional regulation by acting as competing endogenous RNAs (ceRNAs), sharing miRNA recognition elements (MREs) and derepressing all target genes in the respective miRNA family^39^. However, notably, most circRNAs in mammals are expressed at low levels and rarely show enrichment of binding sites for their target miRNAs^40^; thus, the role of the ceRNA hypothesis in the biological function of circRNAs is uncertain^7^. circRNAs may interact with different transcription factors and proteins to form specific RNPs and influence their intrinsic function^41^. Du et al. revealed that ectopic expression of circ-Foxo3 inhibits cell cycle progression via formation of the circFoxo3-p21-CDK2 ternary complex and reduces the level of CDK2, which can accelerate cell cycle entry by interacting with cyclin A and cyclin E^42^.

Our mechanistic study showed that circXPO1 serves as an oncogene and binds competitively to IGF2BP1, mediating the RNA stability and translation of the CTNNB1 protein as a result of this interaction. As an essential onco-foetal protein, IGF2BP1 has been demonstrated to stabilize its target transcripts and mediate translation during multiple biological processes via the formation of particular mRNP granules^43^. IGF2BP1 was previously shown to be localized predominantly in the cytoplasm^44^, similar to circXPO1, which was proven by our subcellular fractionation assay. Moreover, as shown by qRT-PCR and western blotting, silencing and overexpression of circXPO1 markedly modulated the nucleic acid and protein levels of IGF2BP1 target factors but did not impact the IGF2BP1 status. Encouragingly, verification of IGF2BP1 as a biological binding partner of circXPO1 further supports the characteristics of IGF2BP1 in transcriptional regulation, extending the actions of these RBPs beyond their conventional functions in mRNA processing.

Another meaningful finding in our study was that circXPO1 increases CTNNB1 (β-Catenin) expression in LUAD cells by interacting with IGF2BP1 and regulating the transcriptional stability of CTNNB1. Akiri et al reported that approximately 50% of human NSCLC cell lines exhibit excessive activation of the Wnt/β-Catenin signalling pathway^43^, and CTNNB1 was found to be expressed in 51% of resected LUAD tumor samples^45^. Although CTNNB1 is an essential regulator of tumor processes, our study provides the first evidence that its mRNA stability and protein expression are regulated by a circRNA. This finding is powerful proof of the indispensable roles of circRNAs in tumors.

Furthermore, circRNAs are promising ideal biomarkers for a variety of diseases because of their stable structure, long half-life without degradation in exosomes and distinct expression models^7^. Herein, PDX models were employed to investigate circXPO1, and we showed that intratumoural injection of siRNA-circXPO1 inhibited LUAD growth in vivo, indicating that circXPO1 might be a therapeutic target in LUAD.

In summary, we identified a novel circRNA, circXPO1, that is overexpressed in LUAD tissues possibly due to amplification of the XPO1 gene. circXPO1 binds with IGF2BP1 and subsequently promotes the stabilization of CTNNB1 mRNA. Our results add new knowledge to the oncogenic role of XPO1 and suggests that noncoding transcripts of coding genes might not be overlooked.

## Supplementary information

Supplementary legends

Supplementary Tables

Supplementary Figure 1

Supplementary Figure 2

Supplementary Figure 3

Supplementary Figure 4
