# Supplementary material for: A novel circular RNA, circXPO1, promotes lung adenocarcinoma progression by interacting with IGF2BP1
Source: Cell Death Dis. 2020 Dec 2;11(12):1031. doi: 10.1038/s41419-020-03237-8 (PMC7710735; doi:10.1038/s41419-020-03237-8)
Supplement: Supplementary file 1 — Supplementary legends [file 41419_2020_3237_MOESM1_ESM.docx]

**Supplementary legends**

**Table S1**. PCR primers and siRNA sequences

**Table S2**. The antibodies for Western blotting

**Table S3**. Baseline of analyzed lung adenocarcinoma patients and correlation between circXPO1 expression and clinical characteristics (n=76)

**Table S4**. Correlation between circXPO1 expression and clinical characteristics and survival (n=61)

**Table S5**. Mass spectrometry results of MS2 pull-down assay

**Figure S1**. circXPO1 inhibits apoptosis and promotes metastasis of LUAD cells

TUNEL assay (a), wound healing assay (b) and transwell assay (c) in A549 cells upon silence and overexpression of circXPO1. Protein expression of PCNA, E-cadherin, and Vimentin upon circXPO1 knockdown and overexpression in A549 cells (d). (**P*<0.01, ***P < 0.01* with a two-tailed *t* test)

**Figure S2**. CTNNB1 mRNA stability in SPC-A1 cells.

qRT-PCR was used to measure the expression levels of CTNNB1 after actinomycin D treatment in the context of circXPO1 overexpression (a) and silence (b) in SPC-A1 cells. **P*<0.01, ***P < 0.01* with Mann-Whitney U test.

**Figure S3**. Rescue experiments in SPC-A1 cells.

Design of rescue experiments (a). Colony formation (b) and MTT assay (c) in SPC-A1 cells. *P* values were calculated by non-paired student tests. **P* < 0.05, ***P* < 0.01. The error bars indicate the standard deviations.

**Figure S4**. IHC Staining of xenograft tumour

Ki-67, CTNNB1, and Cyclin-D1 were stained in xenograft tumour tissues (*the scale bar represents 200 µm*).
