# Supplementary figures and images for: A novel circular RNA, circXPO1, promotes lung adenocarcinoma progression by interacting with IGF2BP1
Source: Cell Death Dis. 2020 Dec 2;11(12):1031. doi: 10.1038/s41419-020-03237-8 (PMC7710735; doi:10.1038/s41419-020-03237-8)

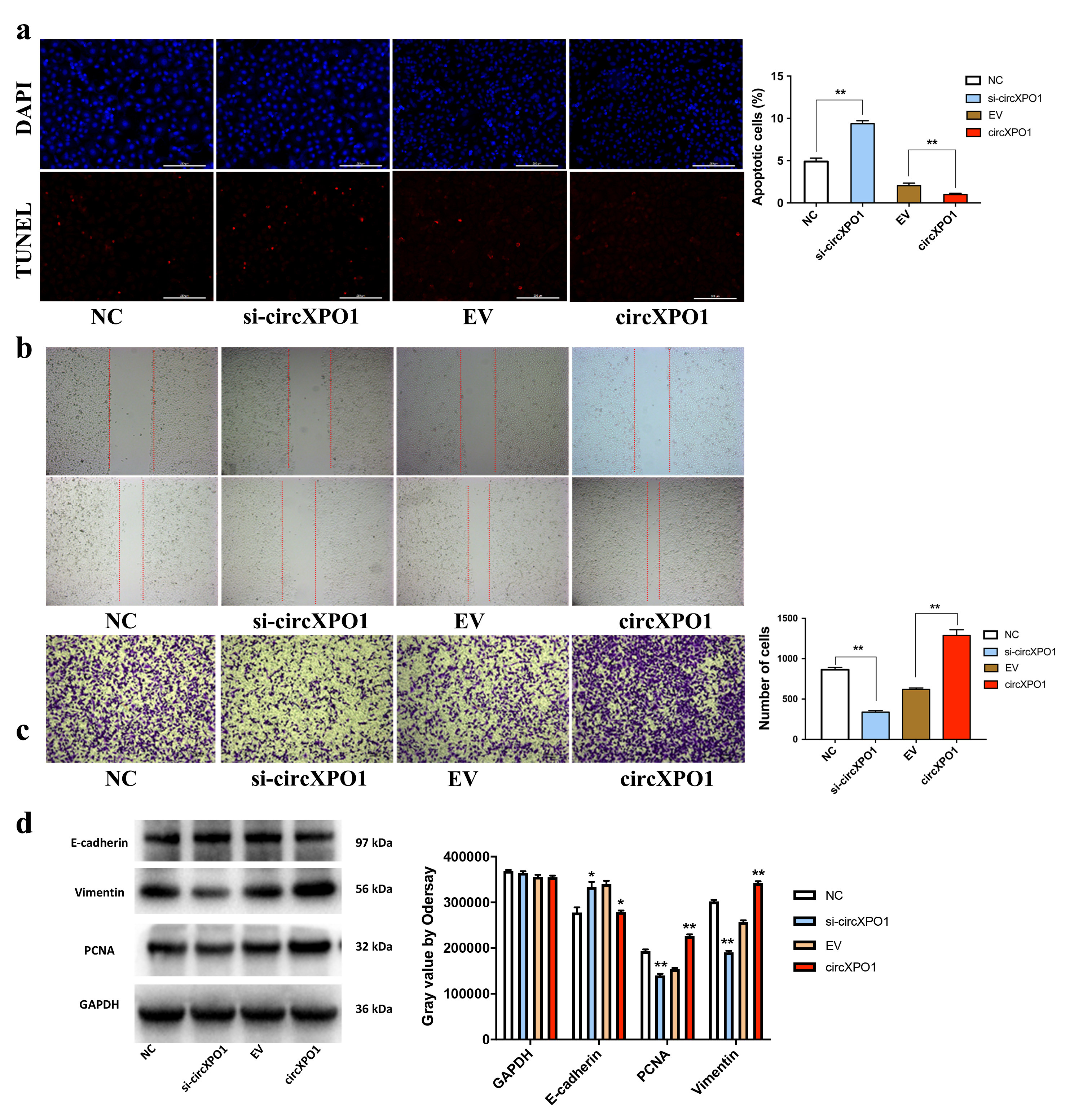

Supplement: Supplementary file 3 — Supplementary Figure 1 [file 41419_2020_3237_MOESM3_ESM.tif]

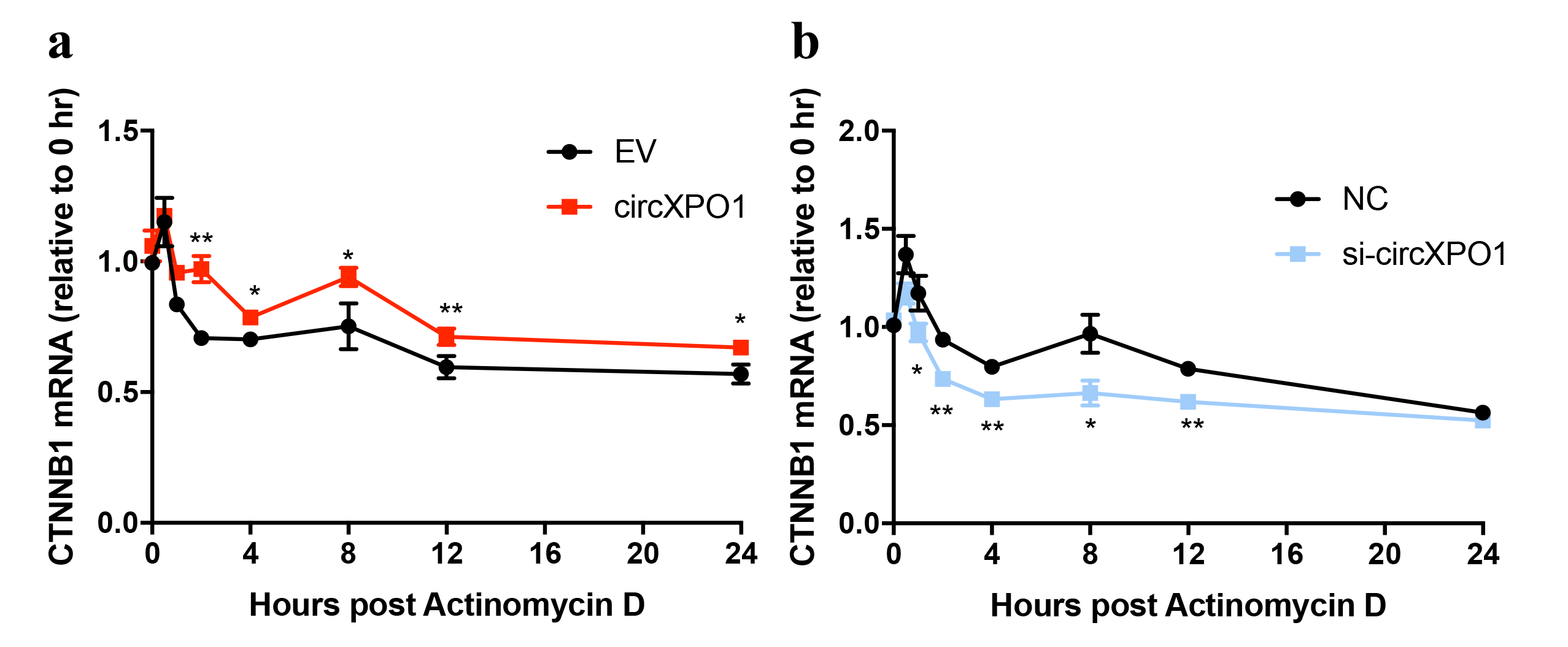

Supplement: Supplementary file 4 — Supplementary Figure 2 [file 41419_2020_3237_MOESM4_ESM.tif]

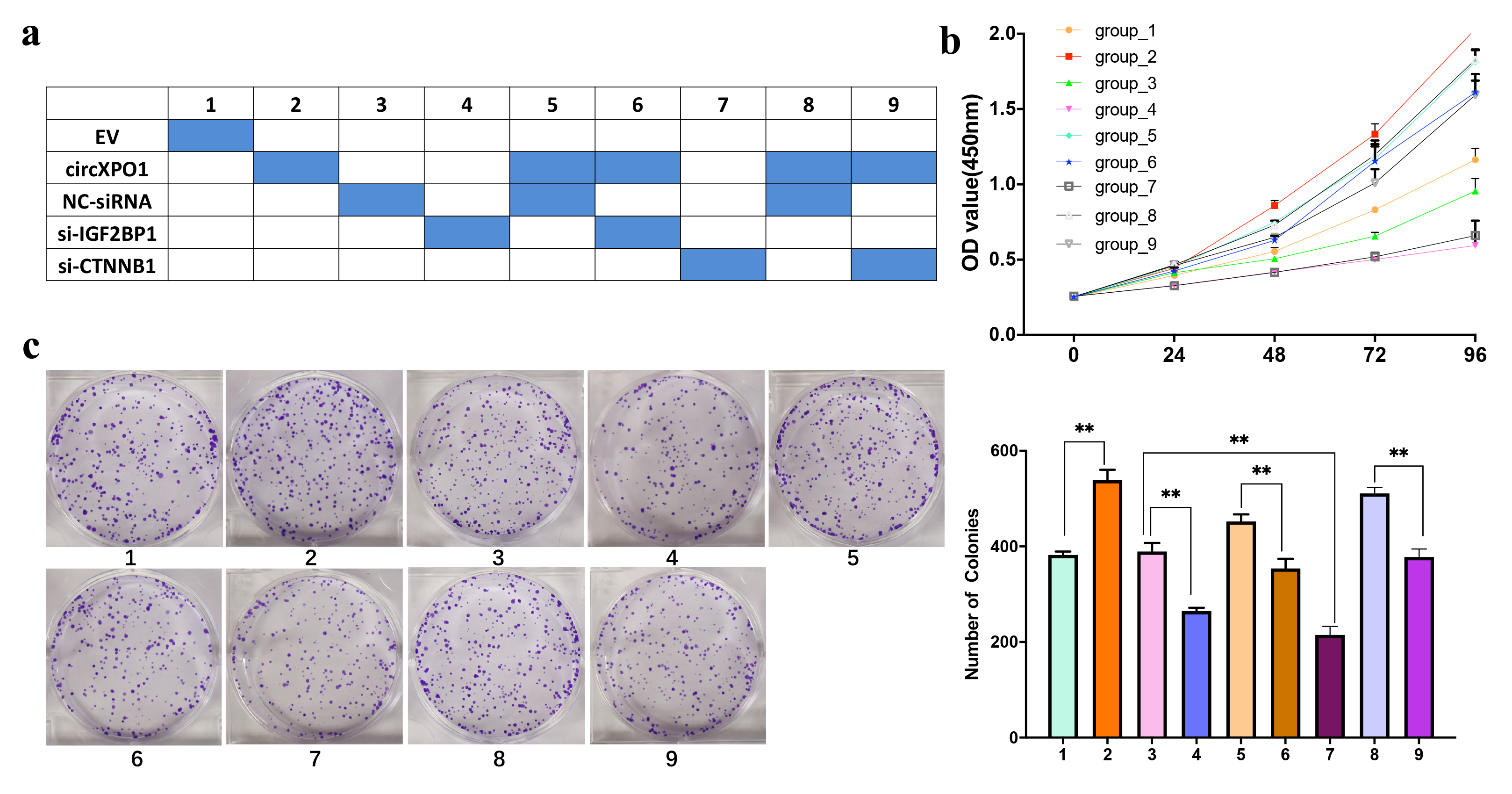

Supplement: Supplementary file 5 — Supplementary Figure 3 [file 41419_2020_3237_MOESM5_ESM.tif]

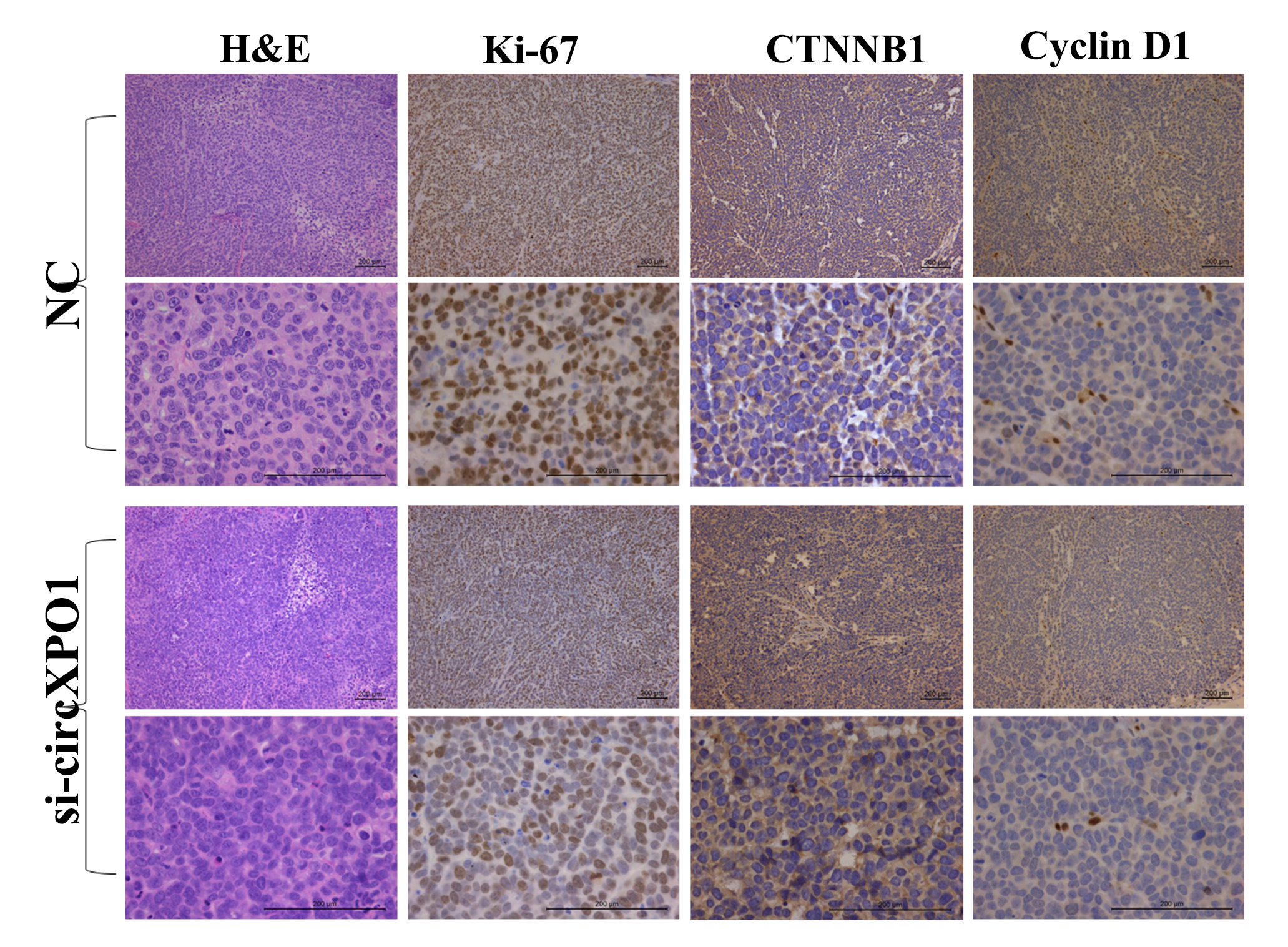

Supplement: Supplementary file 6 — Supplementary Figure 4 [file 41419_2020_3237_MOESM6_ESM.tif]
